# Correction: Kasza et al. New, Spherical Solutions of Non-Relativistic, Dissipative Hydrodynamics. *Entropy* 2022, *24*, 514

**DOI:** 10.3390/e24060821

**Published:** 2022-06-13

**Authors:** Gábor Kasza, László P. Csernai, Tamás Csörgő

**Affiliations:** 1 Wigner Research Centre for Physics, H-1525 Budapest, Hungary; kasza.gabor@wigner.hu; 2 MATE Institute of Technology KRC, Mátrai út 36, H-3200 Gyöngyös, Hungary; 3 Department of Atomic Physics, Eötvös Loránd University, Pázmány P. 1/A, H-1118 Budapest, Hungary; 4 Department of Physics and Technology, University of Bergen, Allégt. 55, 5007 Bergen, Norway; laszlo.csernai@uib.no; 5 Frankfurt Institute for Advanced Studies, 60438 Frankfurt, Germany

## 1. Change in Main Body Paragraphs

In the original publication [1], the authors identified an unfortunate typo, a missing factor of 3, which appeared due to the divergence of a spherically symmetric Hubble flow field given by Equation (15). This missing factor affected Equation (26) at two different places and Equation (43) at one location. Namely, the corrected form of Equation (26) for 3 spatial dimensions reads as the following:

$$R\ddot{R}=C_{E}\frac{T}{m}\left(1-3\frac{\zeta_{0}}{p_{0}}\frac{\dot{R}}{R}\right)=C_{E}f_{T}\left(t\right)\frac{T_{0}}{m}\left(1-3\frac{\zeta_{0}}{p_{0}}\frac{\dot{R}}{R}\right).$$

The corrected form of Equation (43) for *d = 3* spatial dimensions reads as follows:

$$R\ddot{R}=g_{T}\left(t\right)\frac{T_{0}}{m}{\left(\frac{R_{0}}{R}\right)}^{\frac{d}{\kappa_{0}}}\left(1-3\frac{\zeta_{0}}{p_{0}}\frac{\dot{R}}{R}\right).$$

## 2. Change in Figures

The authors propagated the numerical effects of this missing factor of 3 in Equations (26) and (43) and fixed Figure 3 and Figure 4 to reflect these numerical corrections. Fortunately, this typo did not affect the text of the manuscript. The corrected figures appears below.

The authors would like to apologize for any inconvenience caused to the readers by these changes. The original publication has also been updated.

## Figures and Tables

**Figure 3:**
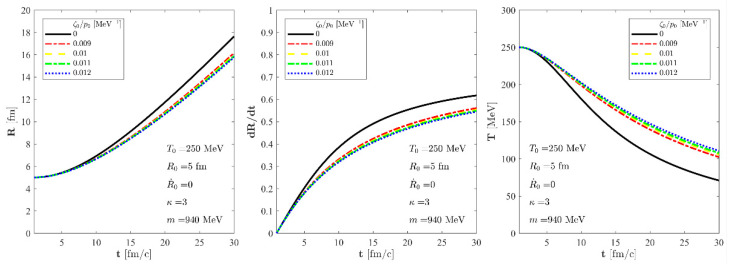
The evolution of the R(t) scale of the fireball (**left**), its time derivative $\dot{R}\left(t\right)$ (**center**), and the temperature (**right**) as a function of time for an exact solution of the non-relativistic Navier-Stokes equations for fixed $T_{0}\;$ = 250 MeV, $R_{0}\;$ = 5 fm and ${\dot{R}}_{0}\;$ = 0 initial parameters. We assume a nuclear fluid here with *m* = 940 MeV particle mass and a constant, temperature-independent *κ* parameter: *κ* = 3. The solid black line stands for a perfect fluid solution, while the dashed blue, the dotted–dashed green, the dashed yellow, and the dotted–dashed red lines correspond to our new viscous solution of non-relativistic Navier-Stokes equations for different values of $\zeta_{0}/p_{0}$ but for the same initial conditions.

**Figure 4:**
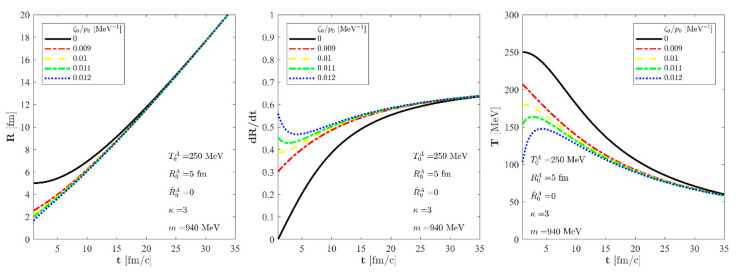
The evolution of the R(t) scale of the fireball (**left**), the $\dot{R}\left(t\right)$ scale velocity (**center**), and the temperature (**right**) as a function of time for the solution of the non-relativistic Navier-Stokes equations for $T_{0}^{A\;}$ = 250 MeV, $R_{0}^{A}$ = 5 fm, and ${\dot{R}}_{0}^{A}\;$ = 0 initial parameters, utilising an *m* = 940 MeV for the particle mass and a constant, temperature-independent *κ* = 3. The solid black line stands for a perfect fluid solution, and this perfect fluid curve labelled by zero bulk viscosity is approached by each of the shown exact viscous solutions asymptotically, T(t) ~ $T_{A}\left(t\right)$. The dashed blue, the dotted–dashed green, the dashed yellow, and the dotted–dashed red lines correspond to our new viscous solution of non-relativistic Navier-Stokes equations for different values of $\zeta_{0}/p_{0}$, but for the same asymptotic solutions.
